# P305: Infectious risk perception and knowledge of medical dentists hygiene of the region Bizerte – Tunisia

**DOI:** 10.1186/2047-2994-2-S1-P305

**Published:** 2013-06-20

**Authors:** K Mrabet, S Arfaoui, H Kammoun

**Affiliations:** 1 Regional Hospital Menzel Bourguiba, Menzel Bourguiba , Bizerte, Tunisia; 2 Faculty of Dental Medicine, Monastir, Bizerte, Tunisia; 3 Regional Direction of Health Bizerte, Bizerte, Tunisia

## Introduction

The risk of infection control in dental health care settings must be an institutional priority, it is an ethical, moral and legal obligation to provide care in hygienic conditions so as to reduce the risk of infection in acts of dental care. The problem that arises in the Tunisian context is primarily a misperception of risk on the one hand, and non-compliance with standard and supplementary precautions.

## Objectives

We conducted a survey of dentists in the area Bizerte to understand the perception of dentists of infectious risks associated with dental care and evaluate their knowledge of prevention of such risks.

## Methods

A self-administered questionnaire was used for data collection. The number of explored items is 43 divided into three categories: i) perception of risk of infection in the dental office ii) general prevention of healthcare associated infections in dental care iii) accidental exposure to blood.

## Results

The number of respondents to the questionnaire assessment of knowledge and risk perception is 62 dentists (18 in the public sector and 44 in the private sector). Overall, the level of knowledge of interviewed dentists , proved quite satisfactory whether old or new to the practice of the profession, whether in the public or private sector with disparities between different explored items.

## Conclusion

Considering the results of this study, the first of its kind to our knowledge in the Bizerte region, we issue the following recommendations to improve the hygiene and safety of dental care in the region : dental awareness on nosocomial infection risk, training in health and safety of dental development of procedures and datasheets health and safety of dental care.

## Disclosure of interest

None declared.

